# Cardiorespiratory fitness, genetic susceptibility, and the risk of chronic obstructive pulmonary disease: findings from observational and two-sample bidirectional Mendelian randomisation analyses

**DOI:** 10.1186/s12916-026-04854-4

**Published:** 2026-05-21

**Authors:** Ziyuan Chen, Paul James Collings, Qiaoxin Shi, Shiu Lun Au Yeung, Shan Luo, Chenxi Li, Tomas Gonzales, Soren Brage, Youngwon Kim

**Affiliations:** 1 School of Public Health, LKS Faculty of Medicine, The University of Hong Kong, Hong Kong Special Administrative Region, Pokfulam, China; 2 MRC Epidemiology Unit, School of Clinical Medicine, University of Cambridge, Cambridge, UK

**Keywords:** Cardiorespiratory fitness, Genetics, Chronic obstructive pulmonary disease, Mendelian randomisation, Gene-environment interaction

## Abstract

**Background:**

Cardiorespiratory fitness (CRF) is a common risk factor for cardiometabolic diseases, but the causal relation of CRF with chronic obstructive pulmonary disease (COPD) and its interplay with genetic risk remain unknown. We integrated genetic susceptibility and causal inference methods to evaluate the protective roles of CRF in COPD development.

**Methods:**

We included 68,288 White British individuals from the UK Biobank (aged 40–79 years) without prevalent COPD at the baseline (2006–13). CRF was assessed using heart rate responses to submaximal bike tests. Genetic risk for COPD was quantified using a polygenic risk score constructed from 71 uncorrelated single nucleotide polymorphisms. Cox regression was used to estimate the hazard of COPD. Causal inference was evaluated via two-sample Mendelian randomisation (MR). Potential reverse causation was assessed using a bidirectional MR.

**Results:**

Within an MR framework, we found that higher genetically predicted CRF is causally associated with lower risk of COPD. Observational analysis found: (1) compared with low CRF (bottom tertile), hazard ratios (95% CI) of COPD were 0.80 (0.72–0.89) and 0.71 (0.64–0.80) for medium and high CRF, respectively; (2) compared to the high CRF-low genetic risk group, COPD hazards were higher for individuals who had medium or high genetic risk combined with low or medium CRF but not for those who had medium genetic risk but high CRF; (3) low CRF combined with any levels of genetic risk showed consistently higher COPD hazards relative to high CRF and low genetic risk combination.

**Conclusions:**

Being more aerobically fit may prevent or delay the onset of COPD. Improving CRF has the potential to attenuate the increased risk of COPD associated with elevated genetic risk. Public health initiatives should prioritise making measurable improvements in cardiorespiratory fitness (beyond merely being active or exercising more) as a promising intervention target for reducing the risk of COPD.

**Supplementary Information:**

The online version contains supplementary material available at 10.1186/s12916-026-04854-4.

## Background

Chronic obstructive pulmonary disease (COPD), a life-threatening lung disease characterised by chronic respiratory symptoms and airflow limitation [1], is the fourth leading cause of chronic morbidity and death worldwide [2]. Its rising incidence makes COPD a critical global public health challenge, accounting for 5% of global deaths, with over 3 million deaths attributable to COPD in 2021 [2]. These alarming statistics translate into a significant economic burden on healthcare systems, as evidenced by the rise in European COPD-related costs from €38.6 to €48.4 billion over a decade [3]. Hence, identifying modifiable risk factors for primary prevention of COPD is urgent.

Tobacco smoking is established as the principal risk factor for COPD [4]. However, 25–45% of COPD cases occur in non-smokers, highlighting substantial heterogeneity in COPD susceptibility that extends beyond tobacco use [5, 6]. These observations indicate complex pathogenesis beyond traditional environmental exposures (e.g. smoking and air pollutants), highlighting the importance of other risk factors, such as genetic architecture [7].

Heritability estimates for COPD and COPD-related lung dysfunction ranged from 40 to 50% [8, 9]. Genome-wide association studies (GWAS) have identified multiple representative genes (e.g. CHRNA3/5, FAM13A, IREB2) for COPD [10–12]. Whilst monogenic variations contribute to disease risk, they often explain only a small fraction of inherited susceptibility [13]. Polygenic risk scores (PRS) can aggregate the effects of various single nucleotide polymorphisms (SNPs) to quantify individuals’ genetic predisposition to COPD. With advances in genomic sciences, GWASs have identified numerous risk-associated SNPs that can be integrated into a PRS [11, 14], enabling better predictive power on COPD risk (AUC increased from 0.58 to 0.68) [8]. However, unexplained variability in PRS points to potential environmental contributions.

Physical activity and exercise have been increasingly recognised as pivotal factors in the pathogenesis and management of COPD in recent years [15–17]. A key underlying mechanism is its role in enhancing cardiorespiratory fitness (CRF). CRF, which reflects the capacity of the circulatory and respiratory systems to supply oxygen to skeletal muscle, is a vital indicator of cardiopulmonary function [18] and a strong marker of healthy longevity [19]. Observational studies have reported inverse CRF-COPD relationships [20–23], but mostly had limited sample sizes (*N*≈2000–4000 participants) or sex- and age-specific samples. However, no prior study has accounted for individuals’ unique genetic susceptibility when examining the role of CRF, nor established the causality between CRF and COPD.

In this study, we aimed to investigate whether CRF modifies the risk of COPD across different levels of genetic susceptibility, and to assess the causal relationship between CRF and COPD risk using a two-sample Mendelian randomisation (MR) approach as previously demonstrated for other disease endpoints [24, 25].

## Methods

### Study population

This study used data from the UK biobank cohort [26, 27], with 502,625 adults aged 40–69 years recruited across the UK during 2006–2010. During this baseline visit, participants underwent a comprehensive set of data collection procedures including touchscreen questionnaires, physical exams, and blood sampling for biochemical and genetic analyses. A repeat-assessment was conducted between 2012 and 2013. Across the baseline and repeat-assessments, 97,950 CRF tests were administrated via sub-maximal ergometry bike (75,087 from baseline and 17,109 from the repeat-measures visit). Two thousand eight hundred seventy-seven participants performed the bike tests on both visits whilst the vast majority performed bike tests once. To maximise the number of incident COPD cases, the earliest CRF record was selected for participants with repeated measures of CRF.

This analysis included White British participants without prevalent COPD at the analytic baseline. Participants were included only if they had a complete data profile, including at least one CRF measure, QC-passed genotyped data, and complete covariate information. The final analytical sample comprised 68,288 individuals (Additional file 1: Fig. S1). The UK Biobank study was approved by Northwest Multi-Centre Research Ethics Committee with participant consent.

### Exposures

#### Cardiorespiratory fitness

In UK Biobank, CRF tests were conducted on a submaximal cycle ergometer, with VO_2_max estimated using validated estimation algorithms calibrated against gold-standard maximal CRF tests [28]. For participants’ safety and protocol appropriateness, protocols were tailored for each participant based on health risk profile, such that individuals with lower exercise capacity or higher risk for exercise-related complications were assigned to a lower workload protocol. This resulted in 11 different test protocols for both men and women. Full methodological details are described elsewhere [28]. CRF estimates were standardised within sex and age strata (< 50, 50–54, 55–59, 60–64, and ≥ 65 years) [29], and categorised into tertiles (high, medium, low CRF groups).

#### Genetic instrument for cardiorespiratory fitness

Detailed information on GWAS for CRF is described elsewhere [25]. In brief, multiple SNPs associated with CRF (*P* < 5 × 10^−8^) were discovered based on 69,416 UK Biobank participants who completed the CRF assessment. In total, 14 independent genome-wide significant variants for CRF (*P* < 5 × 10^−8^) were included to construct the instrument for CRF. Genetic instruments for CRF were derived after the adjustment of recruitment age, sex, genotyping array, and the first 10 principal components in the GWAS [25].

#### Genetic risk for chronic obstructive pulmonary disease

In the UK Biobank, genotyping was performed using two genetic testing arrays (UK Biobank Axiom Array and UK BiLEVE Axiom Array), the details are described elsewhere [26]. To calculate genetic risk for COPD, we utilised a PRS derived from genome-wide significant (*P*-value < 5 × 10^−8^) SNPs associated with COPD/lung function (via FEV1 or FEV1/FVC) [11]. We retained 71 uncorrelated SNPs after clumping (linkage disequilibrium *r*^2^ < 0.001) (Additional file 1: Table S1). As the COPD GWAS analysed data involving UK Biobank participants, we calculated an unweighted PRS for COPD to prevent over-fitting [30]. PRS was computed by summing the number of risk-increasing alleles across all SNPs [31]. Participants were categorised into tertiles (low, medium, high groups).

#### Genetic instrument for chronic obstructive pulmonary disease

We obtained the genetic instruments for COPD from the largest and most recent meta-analysis of GWAS summary statistics from a Global Biobank study including data from 29 biobanks across four continents. The current analyses were restricted to use European-specific effect estimates (*N* = 995,917: 58,559 cases, 937,358 controls). Data source: https://www.ebi.ac.uk/gwas/studies/GCST90399694 [32]. We excluded instruments which were in high linkage disequilibrium (LD) with other instruments (*r*^2^ < 0.001) and consequently identified 21 independent variants for COPD at genome-wide significant level (*P* < 5 × 10^−8^). After we harmonised for the genetic instruments in the outcome datasets, 19 variants for COPD (as the exposure) were retained for the bidirectional analyses.

### Outcomes

#### COPD ascertainment

In the observational analyses, we identified incident COPD cases between the date of completion of the CRF assessment and the last available follow-up date (July 4, 2024, for England/Wales; July 2, 2024, for Scotland) via hospital admission records and death registrations using diagnosis codes from ICD-9 (491, 492 and 496) and ICD-10 (J43-J44). Follow-up time was calculated from the date of the CRF assessment until the earliest occurrence of COPD diagnosis, death, loss to follow-up, or the censoring date. Individuals who developed the COPD cases within the first 2 years of follow-up were excluded from the analyses.

#### Genetic associations with chronic obstructive pulmonary disease

For the MR analyses, genetic associations on COPD were extracted from the largest and most recent meta-analysis of GWAS summary statistics from a Global Biobank study including data from multiple continental biobanks [32]. In the current analyses, we used European-specific effect estimates (*N* = 995,917: 58,559 cases, 937,358 controls. Data source: https://www.ebi.ac.uk/gwas/studies/GCST90399694.

#### Genetic associations with cardiorespiratory fitness

For the bidirectional analyses, genetic associations on CRF were extracted from the recent GWAS summary statistics conducted in UK Biobank [33]. We used the effect estimates from 70,783 European ancestry individuals in the current analyses (Data source: https://www.ebi.ac.uk/gwas/studies/GCST90093316).

### Potential confounders

The following variables were considered as confounders, given their plausible associations with the exposures and outcome, whilst not functioning as mediators: sex (male/female), smoking behaviour (never/previous/current), alcohol consumption frequency (never/previous/current [with current drinkers further stratified into < 3 times/week and ≥ 3 times/week]), self-reported employment status (unemployed/employed/retired), the Townsend Deprivation Index (a postcode-derived measure of socioeconomic disadvantage), air-pollution exposures (residential levels of PM₂.₅, PM₁₀, and nitrogen dioxide), muscle strength (assessed via grip strength), body mass index, history of diabetes, and history of hypertension, history of asthma, genotyping array (UK Biobank Axiom Array and UK BiLEVE Axiom Array), and the first twenty principal components of ancestry.

### Statistical analysis

We presented descriptive statistics as means with standard deviations for continuous variables and percentages for categorical variables. Multivariable Cox regression models were fit with age modelled as the underlying timescale to estimate hazard ratios (HRs) and 95% confidence intervals (CIs) for the risk of COPD. Cumulative hazards curves were plotted to visually represent the total accumulated risk of COPD across the age spectrum within PRS categories, adjusted for sex, genotype arrays, and the first twenty genetic principal components.

To quantify the observational associations of genetic risk with incident COPD, we adjusted Cox regression models for age (as the underlying timescale), sex, genotyping array and first twenty principal components of ancestry (calibrating for population stratification). In the models using CRF as the exposure, two sequential models were built: Model 1 using age as the timescale and adjusted for potential confounders. Model 2 (main model) was additionally adjusted for PRS, genotyping array and first twenty principal components of ancestry in addition to confounders.

Interactions (multiplicative [likelihood ratio tests] and additive interaction [relative excess risk due to interaction]) between the categories of PRS (high, medium, low genetic risk) and CRF (high, medium, low) on incident COPD were investigated using Cox regression models, adjusted for potential confounders. Considering baseline COPD risk that varies across genetic risk categories, we built a model to examine the joint association between CRF and COPD genetic risk, where a total of nine joint PRS-CRF groups (three categories of PRS × three groups of CRF) were created with the highest CRF-lowest genetic risk combination as the reference. The joint analysis facilitates the depiction of the patterns on how CRF may interplay with COPD genetic risk in shaping the risk of COPD. Estimates of 10-year absolute risk of COPD were quantified for each combined category of genetic risk and CRF using Cox regression models, with follow-up time as the underlying time scale, adjusted for age, sex, genotyping arrays, and the first twenty genetic principal components.

In the MR analyses, inverse variance weighted (IVW) analysis is the primary method to assess the potential causal effect of CRF on COPD. This instrumental variable analysis is less biased to residual confounding and reverse causation [34]. It relies on three core assumptions: relevance (the instruments must be associated with exposure), independence (the instruments are not related to confounding factors) and exclusion restriction (the instruments affect the outcome only through the exposure). We evaluated instruments’ strength (*F*-statistic) [35], heterogeneity within instrument-specific Wald Ratio (Cochran’s *Q* test) [36], and overall horizontal pleiotropy (intercepts from MR-Egger regression) [37]. A series of additional statistical tests were performed to examine the robustness of the primary MR results (i.e. using weighted median [38], and MR-robust adjusted profile score methods [39] to account for potential bias arising from violation of the IV assumptions [40]). MR-PRESSO (Pleiotropy Residual Sum and Outlier) was used to correct outliers reflecting potential pleiotropic bias [41].

Given that the unidirectional MR design may not address the possibility of reverse causation where predisposition to COPD might lead to variation in CRF, we performed a bidirectional Mendelian randomisation analysis using genetic predictors of COPD as instruments [32]. The IVW method was used as the primary analysis to evaluate the causal effect of COPD on CRF and explicitly test this potential reverse pathway.

Several sensitivity analyses were performed: (1) to reduce the potential for reverse causation, we excluded COPD cases diagnosed within the first 4 years of follow-up; (2) we further adjusted statistical models for behavioural factors including moderate and vigorous physical activity (MVPA, minute/day), TV viewing time (hours/day), and sleep duration (< 7 h/day, 7–9 h/day, > 9 h/day) to examine the observational CRF-COPD associations, as they may also be confounders rather than precursors to CRF; (3) to reduce potential misclassification in self-reported ethnicity, we repeated analyses by including genetically defined White British ancestry via principal component analysis of genomic data; (4) to reduce the potential misclassification of baseline COPD status, we conducted further confirmation via spirometry in all participants, excluding individuals with airflow obstruction (defined as a FEV1/FVC ratio < 0.70) at baseline; (5) to evaluate the robustness of our findings against a broader case definition, we repeated the analysis estimating the associations of CRF and genetic risk with UK Biobank algorithmically defined COPD (Data-fields 42,016); and (6) for the MR analysis exploring the CRF-COPD association, another genetic instrument comprising 160 genetic variants was identified as a stronger proxy of CRF which explained a higher proportion of phenotypic variance in CRF compared with the instrument based on 14 SNPs in the main MR analysis [25]. However, we found 66% of variants were identified as weak (*F* statistic < 10). Therefore, we further constructed a genetic instrument for CRF using 160 independent SNPs [25]. For all Cox models, we verified adherence to the proportional hazards assumption using Schoenfeld residuals and log–log survival plots. PRS were computed using Plink 2.0 software. Observational Analyses were performed in STATA (version 17.0; StataCorp MP, College Station, TX), MR analyses were performed using the TwoSampleMR packages in R version 4.1.0 [42].

## Results

### Observational analysis

The final analytic sample included 68,288 White-British participants (52.6% female, baseline mean age: 58.3 years) free from COPD at baseline. Over an average follow-up of 13.2 years, 1936 incident COPD cases were recorded. Detailed baseline characteristics are provided for the overall sample and within CRF tertiles (see Table 1).

**Table 1:** Baseline characteristics of individuals overall and within tertiles of sex- and age-standardised cardiorespiratory fitness (VO_2max_)

		Tertiles of cardiorespiratory fitness (VO_2max_)		
Variables	All (*N* = 68,288)	Low (*N* = 22,808)	Medium (*N* = 22,723)	High (*N* = 22,757)
Age	58.3 (8.1)	58.3 (8.1)	58.3 (8.1)	58.2 (8.1)
Sex				
Women	52.6	50.9	55.4	51.7
Men	47.3	49.2	44.6	48.3
COPD cases	1936	813	607	516
Follow-up years (average)	13.2 (2.1)	13.1 (2.2)	13.2 (2.0)	13.2 (2.0)
Smoking status				
Never	56.1	55.1	55.8	57.4
Previous	36.0	37.0	36.2	34.7
Current	7.9	7.9	8.0	7.8
Employment				
Unemployed	6.7	7.8	6.2	6.0
Employed	55.0	53.2	55.5	56.4
Retired	38.3	38.8	38.4	37.7
Townsend Deprivation Index	− 1.53 (2.8)	− 1.3 (2.8)	− 1.6 (2.7)	− 1.6 (2.7)
Alcohol consumption				
Never	2.9	3.4	2.8	2.4
Previous	2.9	3.5	2.6	2.5
Current (< 3 times/week)	56.8	60.1	56.0	54.2
Current (≥ 3 times/week)	37.4	32.9	38.6	40.8
Muscle strength (kg)	29.5 (10.6)	29.8 (10.9)	29.2 (10.6)	29.5 (10.3)
VO_2max_ (mL/kg/min)	28.6 (6.7)	23.1 (4.4)	28.2 (3.9)	34.7 (5.6)
Residential air-pollution (micro-g/m^3^)				
PM₂.₅	9.9 (0.9)	9.9 (0.9)	9.9 (0.9)	9.9 (0.9)
PM₁₀	16.3 (1.8)	16.3 (1.8)	16.3 (1.8)	16.3 (1.8)
Nitrogen dioxide	27.3 (7.0)	27.3 (6.8)	27.2 (7.0)	27.4 (7.2)

Continuous variables are reported as means with corresponding standard deviations. Categorical variables are presented as percentages. Abbreviations: *COPD*, chronic obstructive pulmonary disease; *CRF*, cardiorespiratory fitness

**Fig. 1:**
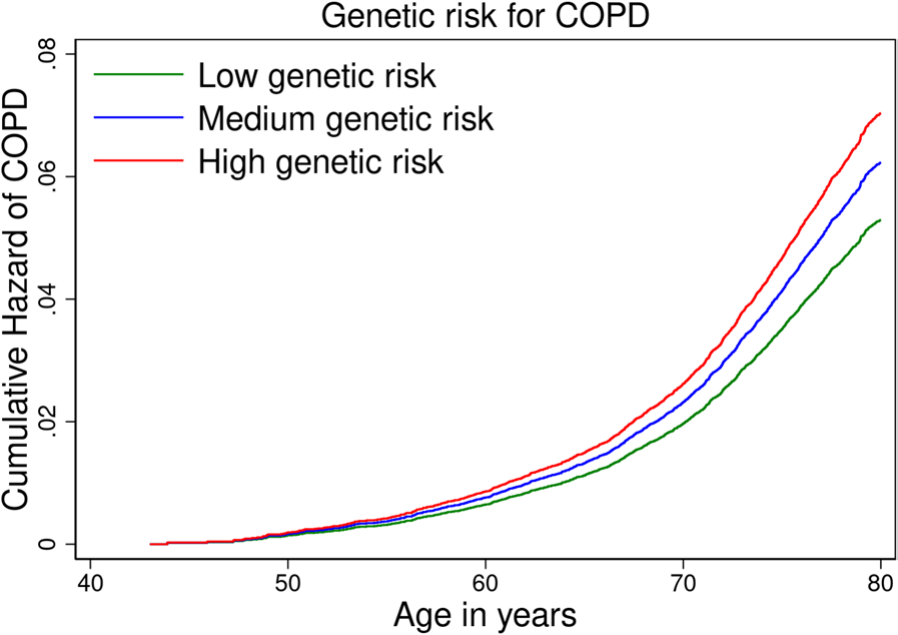
Cumulative hazard rates of chronic obstructive pulmonary disease according to categories of genetic risk. Note: Cox regression models with categories of polygenic risk score as the exposure and incorporating age as the underlying time scale were adjusted for sex, genotyping array, and the first twenty principal components of ancestry. Abbreviation: COPD, chronic obstructive pulmonary disease

Figure 1 illustrates that the cumulative hazards of COPD across the age range were higher in the high genetic risk group compared to the low PRS group. Cox models corroborated this association: high genetic risk (HR: 1.33, 95% CI: 1.19–1.48) and medium genetic risk (HR: 1.18, 95% CI: 1.05–1.32) exhibited 33% and 18% higher hazards of COPD compared to low genetic risk, respectively, after adjusting for sex, genotyping arrays and the first twenty principal components of ancestry (see Table 2).

**Table 2:** Prospective associations of genetic risk and sex- and age-standardised cardiorespiratory fitness with incident chronic obstructive pulmonary disease

			Hazard ratio of COPD	
			(95% confidence intervals)	
Comparisons	No. of participants	No. of events	Model 1	Model 2
Tertiles of polygenic risk				
Score for COPD				
Low (reference)	22,763	548	1.00 (ref)	
Medium	22,769	651	1.18 (1.05–1.32)	
High	22,756	737	1.33 (1.19–1.48)	
PRS-COPD (continuous)	68,288	1936	1.12 (1.07–1.17)	
Tertiles of CRF				
Low (reference)	22,808	813	1.00 (ref)	1.00 (ref)
Medium	22,723	607	0.78 (0.70–0.88)	0.79 (0.70–0.88)
High	22,757	516	0.70 (0.62–0.80)	0.70 (0.62–0.80)
CRF (per 1-SD difference)	68,288	1936	0.86 (0.82–0.91)	0.88 (0.83–0.93)

The analyses were performed based on multivariable Cox proportional hazards models

Model 1: Model for PRS-COPD adjusted for age, sex, genotyping array and the first twenty principal components of genetic ancestry. Model for CRF adjusted for age, sex, muscle strength (assessed via grip strength for both hands), smoking status (never, previous, current), employment (unemployed, employed), Townsend Deprivation Index, alcohol consumption (never, previous, currently < 3 times/week, currently ≥ 3 times/week), body mass index, history of diabetes, history of hypertension, history of asthma, and air-pollution exposure (assessed based on residential levels of PM₂.₅, PM₁₀, and nitrogen dioxide)

Model 2: adjusted for all confounders in model 2 with an additional adjustment for genetic risk, genotyping array and first twenty principal components of genetic variant

Abbreviations: *COPD*, chronic obstructive pulmonary disease; *CRF*, cardiorespiratory fitness; *PRS-COPD*, polygenic risk score for chronic obstructive pulmonary disease

Table 2 further presents the CRF-COPD association. CRF exhibited a strong inverse association with COPD incidence showing that each 1-SD difference in sex- and age-standardised CRF (equivalent to 5.5 mL O_2_/kg/min) was associated with 12% lower risk of COPD independent of putative confounders (see Table 2, Model 2). For categories of CRF, participants with the medium and high CRF had 21% (HR: 0.79, 95% CI: 0.70–0.88) and 30% (HR: 0.70, 95% CI: 0.62–0.80) lower hazards, respectively, compared to the low-CRF reference group. An additional adjustment for genetic susceptibility made no substantive change to the magnitude of the associations in Model 2. These associations remained strong across multiple sensitivity analyses (See Additional file 1: Table S2-S6).

**Fig. 2:**
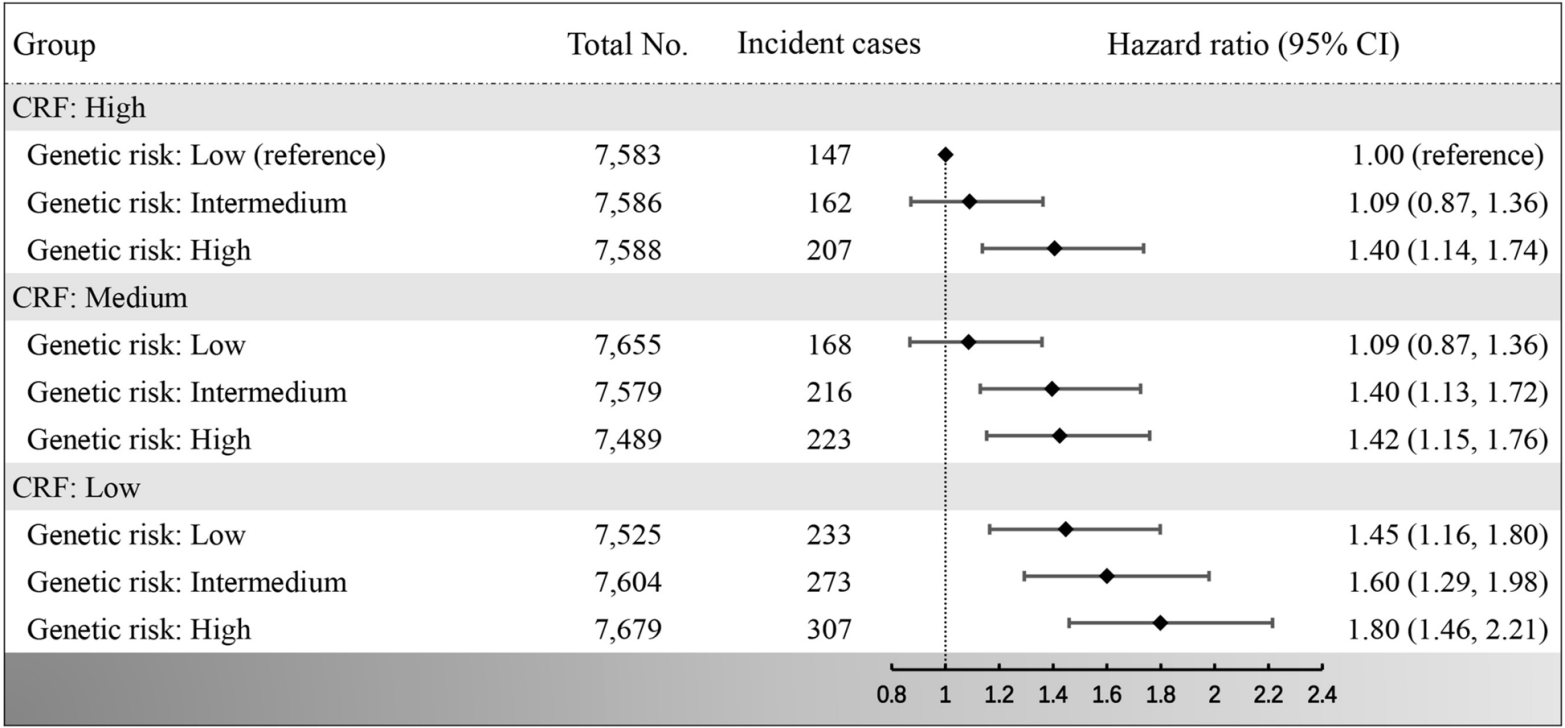
Joint associations of genetic risk, cardiorespiratory fitness with chronic obstructive pulmonary disease. Note: Cox regression model was employed adjusting for age (as underlying time scale), sex, muscle strength (assessed via grip strength for both hands), smoking status (never, previous, current), employment (unemployed, employed), Townsend Deprivation Index, alcohol consumption (never, previous, currently < 3 times/week, currently ≥ 3 times/week), air-pollution exposure (assessed based on residential levels of PM₂.₅, PM₁₀, and nitrogen dioxide), body mass index, history of diabetes, and history of hypertension, genotyping array, and the first twenty principal components of genetic ancestry

Figure 2 shows the joint associations between genetic risk and incidence COPD within CRF levels. Specifically, low CRF combining with any level of genetic risk demonstrated elevated COPD risk compared to high CRF-low genetic risk group (reference group). The highest risk was found in the low CRF-high genetic risk combination (80% higher COPD hazard, 95%CI: 1.46–2.21) when compared to the reference category. Notably, within the high CRF tertile, medium genetic risk had no evidence of associations with COPD but the risk of COPD was greater for high genetic risk, suggesting that high CRF partially attenuated the risk of elevated genetic susceptibility on COPD (see Fig. 2). Meanwhile, we observed that high CRF-high genetic risk combination (HR = 1.40, 95%CI:1.13–1.72) and low CRF-low genetic risk combination (HR = 1.45, 95%CI:1.16–1.80) demonstrated comparable risk relative to the optimal reference group (high CRF with low genetic risk) (see Fig. 2). *P*-values for multiplicative and additive interactions between CRF and genetic risk were 0.35 and 0.75, respectively.

**Fig. 3:**
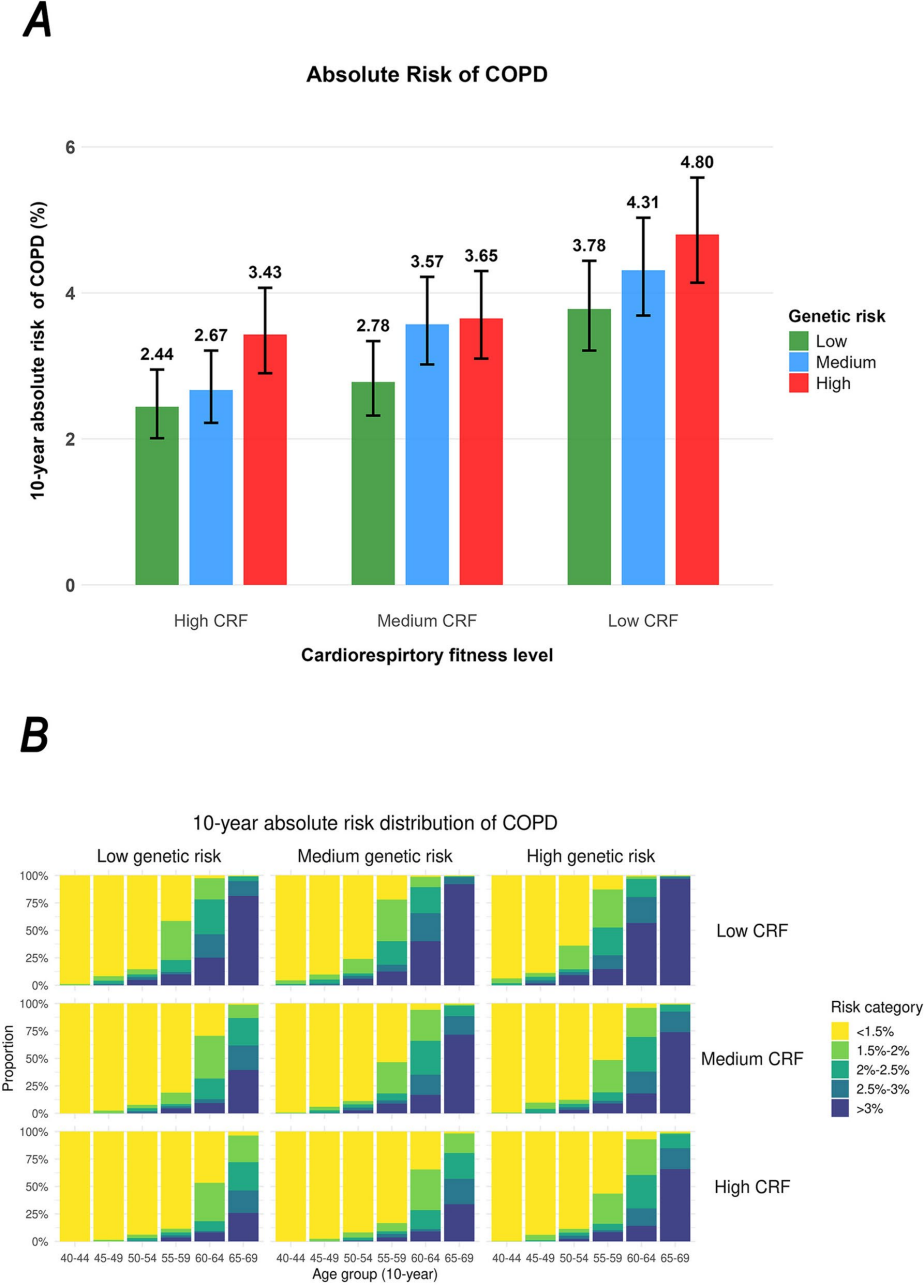
Estimated 10-year absolute risk of chronic obstructive pulmonary disease stratified by categories of genetic risk and cardiorespiratory fitness. Note: **A** 10-year absolute risk of COPD for combined categories of CRF and genetic risk calculated using Cox regression models with follow-up time as the underlying timescale adjusted for sex, genotyping array, and the first twenty principal components. **B** The distribution of 10-year absolute risk estimates of COPD for CRF-genetic risk combined categories across different age ranges. Abbreviation: COPD, chronic obstructive pulmonary disease; CRF, cardiorespiratory fitness

The estimated 10-year absolute risk of COPD varied from 2.44% to 3.43% among individuals with high CRF, whereas those with low CRF exhibited higher risks (3.78% to 4.80%) (see Fig. 3A). Individuals characterised as low CRF and high genetic risk had the highest absolute risk (10-year absolute risk: 4.80%; Fig. 3A). Notably, the 10-year absolute risk of COPD was lower for those at high COPD genetic risk combined with high CRF (3.43%) than for those with low CRF and low genetic risk (3.78%) (see Fig. 3A). Figure 3B illustrates the distribution of COPD 10-year absolute risk estimates across CRF levels, genetic risk categories, and age groups (40–69 years). Across all genetic risk strata, high CRF was associated with a greater likelihood of falling into the risk category with 10-year risk < 1.5%, whilst low CRF was disproportionately associated with larger proportions of higher absolute risk in older adults (10-year risk > 3%) (see Fig. 3B). It is notable that, in the high genetic risk group, low CRF was associated with substantially higher risk particularly among older adults (with > 3% risk predominant in the age group of 65–69 years) (see Fig. 3B).

### Mendelian randomisation analysis

In the Mendelian randomisation framework, 12 of the 14 identified CRF-associated SNPs were retained for analyses after harmonising with COPD GWAS summary statistics. In Table 3, we found strong evidence for the inverse association between CRF and COPD (OR = 0.98 per 1-unit increase in genetically predicted CRF [ml O_2_⋅min^−1^⋅kg^−1^], 95%CI: 0.97–0.99; *P* = 0.021) with no evidence of heterogeneity (Cochran’s *Q P*-value = 0.34) and bias of pleiotropy (MR-Egger Intercept *P*-value = 0.64). A series of additional analyses were performed to examine the robustness such as MR Egger, weighted median models and robust adjusted profile score, where the associations between CRF and COPD were consistent with the main MR analysis (see Table 3), and bias due to weak instruments was unlikely (*F*-statistic > 10 for all SNPs). There is no strong influence of outliers on the primary MR results as identified by the MR-PRESSO method. The genetic instrument of fitness composing 160 SNPs (after excluding the outliers, 94 SNPs were included in both CRF and COPD GWAS results) showed consistent results compared to the main MR analysis (Additional file 1: Table S7).

**Table 3:** Results of Mendelian randomisation of higher genetically predicted cardiorespiratory fitness on chronic obstructive pulmonary disease

Method	Number of SNPs	Odds ratio (95% CI)	*P*-value	Cochran’s *Q P*-value	Egger Intercept *P*-value
Inverse-variance weighted	12	0.98 (0.97–0.99)	**0.021**	0.34	
Weighted median	12	0.99 (0.96–1.007)	0.201		
MR Egger	12	0.99 (0.94–1.04)	0.776		0.64
Robust adjusted profile score	12	0.98 (0.96–0.99)	**0.008**		
MR-PRESSO	12	0.98 (0.96–0.99)	**0.042**		

The effect estimates on cardiorespiratory fitness were extracted from meta-analysis summary statistics among Europeans [25]. The genetic associations for chronic obstructive pulmonary disease were extracted from the meta-analysis of GWAS summary statistics from a Global Biobank study in European ancestry (58,559 cases and 937,358 controls) [32]. Values in bold indicate statistical significance (*p*<0.05)

Table 4 presents the results of the Mendelian randomisation assessing the effect of higher genetically predicted risk of COPD on CRF levels, using genetic instruments for COPD. We observed no evidence on the association of COPD with CRF. This evidence suggests that reverse causation is unlikely to drive the primary relationship between CRF and COPD risk.

**Table 4:** Results of Mendelian randomisation of higher genetically predicted risk of chronic obstructive pulmonary disease on cardiorespiratory fitness

Method	Number of SNPs	Beta (95% CI)	*P*-value	Cochran’s *Q P*-value	Egger Intercept *P*-value
Inverse-variance weighted	19	− 0.003 (− 0.04, 0.04)	0.86	0.45	
Weighted median	19	− 0.003 (− 0.06, 0.06)	0.93		
MR Egger	19	− 0.048 (− 0.16, 0.06)	0.39		0.39
Robust adjusted profile score	19	− 0.007 (− 0.05, 0.04)	0.77		
MR-PRESSO	19	− 0.003 (− 0.06, 0.05)	0.92		

The genetic instruments on chronic obstructive pulmonary disease were extracted from the meta-analysis of GWAS summary statistics from a Global Biobank study in European ancestry (58,559 cases and 937,358 controls) [32]. The genetic associations for cardiorespiratory fitness were extracted from summary statistics among individuals with European ancestry [33]

## Discussion

In this large-scale study with paired CRF and genetic data, we integrated observational and MR analyses to investigate the association between CRF and incident COPD. We provide evidence suggesting a potential causal effect of higher CRF levels on reduced COPD risk, supported by bidirectional MR results arguing against the possibility that this finding is driven by reverse causality. From the observational analysis, we found higher CRF was robustly associated with a reduced risk of COPD, even among individuals with high genetic susceptibility to COPD. Notably, our joint analysis further revealed that high CRF is associated with lower COPD risk even among those with elevated genetic risk.

A prior observational study from our group reported a 10% lower risk of COPD per 3.5 ml O_2_/min/kg difference in CRF but with shorter follow-up compared with the present study [28]. In the current study, similar effect estimates were found where each standard deviation score difference in sex- and age-standardised CRF (equivalent to 5.5 mL O_2_/kg/min) was associated with a 13% lower COPD risk, with the highest CRF group showing a 29% lower hazard versus the lowest. Collectively, our results from the UK align with a Danish cohort study which reported a 31% lower COPD risk among male participants with high CRF relative to low CRF [22]. Another observational study of 2,295 middle-aged and older Finnish men revealed a similar inverse association between CRF and COPD [20]. However, all previous research failed to account for individuals’ genetic risk. Our study advances the evidence base by integrating genetic risk information, demonstrating that CRF, a modifiable physiological trait, is strongly associated with COPD across genetic risk strata in a dose-dependent fashion.

Whilst prior studies focused on CRF mitigating conventional COPD risks (i.e. smoking, obesity) [43, 44], we established its role in attenuating genetically driven COPD susceptibility. We found that high CRF may partially attenuate increased COPD risk associated with elevated genetic risk and could benefit all individuals including those who are genetically vulnerable to COPD. Whilst improved CRF via aerobic training or physical activity [45] is known for its favourable impact on various diseases (i.e. coronary heart disease, type II diabetes, and site-specific cancers [colorectal and breast cancer]) [24, 25, 46], we extend knowledge on the protective role of CRF to COPD under the context of genetic susceptibility. Whilst our MR analysis provided the casual evidence between CRF and COPD, future study is warranted to quantify the degree to which the elevated genetic risk for development of COPD is reduced by improving CRF and elucidate underlying biological mechanisms.

The highest 10-year absolute risk was observed among individuals with low CRF and high genetic risk. The distribution of different levels of absolute risk (as illustrated in Fig. 3B) demonstrates the preventive role of CRF across varying levels of genetic risk and age ranges. For example, individuals with low CRF, even at low genetic risk, had a substantially higher proportion of elevated 10-year COPD risk (> 3%), particularly among older adults (aged over 60 years), compared to individuals with high CRF within the high genetic risk stratum. This age-dependent risk gradient in high genetic risk/low CRF individuals implies targeted fitness interventions in mid-to-late adulthood could mitigate COPD susceptibility in genetically vulnerable populations. Taken together, our findings suggest that individuals with higher CRF tend to possess both lower relative risks and absolute risks of COPD, whilst genetically vulnerable subgroups with low CRF may have more pronounced COPD hazards.

Our study provides novel MR evidence suggesting a causal role of CRF in the risk of COPD, addressing a key gap in the literature given the absence of investigations into this causal pathway. Unlike recent MR studies showing no causal link between MVPA and COPD [47, 48], our finding suggests CRF, an integrative trait representing both input from genetics and physical activity behaviours, appears to better capture protective biological pathways than physical activity. This implies that merely ‘being active or exercise more’ without achieving measurable improvements in CRF may not confer effective benefits against COPD. Thus, CRF may be a superior objective biomarker when evaluating COPD risk, whilst MVPA without CRF improvements may lack physiological adaptations impacting COPD. Although our MR analysis supports causality, the precise biological mechanisms underlying this relationship require further elucidation.

The biological mechanisms linking CRF to COPD pathogenesis, particularly under the context of genetic susceptibility, are yet to be fully understood. We speculate that systemic inflammation, a key driver of COPD pathogenesis [49], may partially explain the association between CRF and disease progression, even in genetically susceptible individuals. The lung, as the primary site of respiratory gas exchange, is chronically exposed to pathogens and pollutants where it harbours numerous resident macrophages that generate inflammatory mediators central to immunologic and inflammatory processes [50, 51]. A cohort study of 2274 middle-aged adults demonstrated that higher CRF attenuated COPD risk associated with elevated inflammation (assessed in the form of high-sensitivity C-reactive protein [hsCRP]). This finding suggests CRF may counteract inflammatory pathways contributing to COPD pathogenesis [23]. The benefits of CRF may also be exerted via the anti-inflammatory effects of high levels of physical activity, which reduces the levels of pro-inflammatory biomarkers such as hsCRP, fibrinogen, and interleukins (IL-1, IL-6) [52, 53]. However, the interplay between the CRF-driven anti-inflammatory cascade and genetic susceptibility on COPD remains unknown. We speculate that CRF may modulate gene-environment interactions (e.g. dampening pro-inflammatory pathways in PRS-high individuals). However, future mechanism studies are warranted. After triangulating observational results and MR evidence for causality, our study suggests that enhancing CRF should be a critical behavioural target for COPD prevention not only in all individuals but also in sub-population groups with potentially greatest benefit, including (1) individuals who are more genetically predisposed with low CRF (the group exhibiting the highest absolute risk) and (2) older adults (age over 60 years) with low CRF who showed a substantial proportion with risk > 3% even at low genetic risk.

A major strength of this study is its integration of genetic susceptibility and causal inference methods, representing the first such effort to evaluate the protective role of cardiorespiratory fitness in chronic pulmonary obstructive disease development. We combined observational and Mendelian randomisation approaches in a large well-characterised cohort with objectively estimated CRF (derived from standardised submaximal exercise tests in the UK Biobank [28]) with genetic data to explore COPD risk. To mitigate reverse causation concerns, early incident COPD cases were excluded (first 2 years of follow-up in primary analyses; 4 years in sensitivity analyses). These methodological advantages collectively provide the most comprehensive evidence to date that CRF is associated with reduced risk of COPD and consistently associated with reduced risk across genetic susceptibility subgroups.

Several limitations should be considered when interpreting our findings. Whilst MR analysis supports a causal relationship between CRF and COPD, the joint associations across genetic risk strata remain observational. Residual confounding may exist despite rigorous covariate adjustment. Definitive causal inference is precluded as the current study is not a randomised controlled trial. Whilst we employed a 2-sample MR design, a small sample overlap was observed (69,416 of participants in the exposure GWAS/393,966 of participants in the outcome GWAS) which may lead to bias towards the confounded association in the presence of weak instrument bias although the *F* statistics suggested this possibility unlikely. For all MR analyses, we cannot fully exclude the possibility of bias arising from violation of IV assumptions, in particular exclusion restriction [34]. Nevertheless, the use of other approaches more resistant to violation of exclusion restriction showed similar findings. This study restricted the analytic sample to individuals of White British ancestry who completed CRF assessments, so findings may limit generalisability to other ethnic groups and those deemed to be at too high acute risk for CRF-test. The results from this study reflect the risk of COPD requiring hospitalisation or causing death and may not be generalisable to mild, outpatient-managed COPD cases.

## Conclusions

We provide novel genetic evidence supporting a causal effect of higher CRF on reduced COPD risk. Our findings suggest that being more aerobically fit can prevent or delay the onset of COPD and has the potential to attenuate the increased risk of COPD associated with elevated genetic risk. The study provides new insights into the potential biological mechanisms linking CRF to COPD pathogenesis. Achieving measurable improvements in CRF, not simply being physically active, should be prioritised as a core approach to COPD prevention, particularly in genetically vulnerable populations.

## Supplementary Information

Additional file 1:Table S1-S7 and Figure S1. Table S1. List of Single-Nucleotide Polymorphisms (SNPs) related to chronic obstructive pulmonary disease. Table S2-S7. Results from sensitivity analyses. Fig S1. Participants’ flow chart. 

## Data Availability

The data that support the findings of this study are available from the UK Biobank (https://www.ukbiobank.ac.uk/) under the application 43,528. UK Biobank data are accessible to researchers with valid licensing approval.

## Abbreviations

CRF: Cardiorespiratory fitness
COPD: Chronic obstructive pulmonary disease
GWAS: Genome-wide association study
SNP: Single nucleotide polymorphisms
PRS: Polygenic risk score
HR: Hazard ratio
OR: Odds ratio
CI: Confidence interval

## References

[1] Agustí A, Celli BR, Criner GJ, Halpin D, Anzueto A, Barnes P, et al. Global initiative for chronic obstructive lung disease 2023 report: GOLD executive summary. Eur Respir J. 2023;61(4):2300239. 36858443 10.1183/13993003.00239-2023 PMC10066569

[2] WHO. Chronic obstructive pulmonary disease (COPD). 2024. [Available from: https://www.who.int/news-room/fact-sheets/detail/chronic-obstructive-pulmonary-disease-(copd)]. Accessed 30 Dec 2025.

[3] Rehman AU, Hassali MAA, Muhammad SA, Harun SN, Shah S, Abbas S. The economic burden of chronic obstructive pulmonary disease (COPD) in Europe: results from a systematic review of the literature. Eur J Health Econ. 2020;21(2):181–94. 31564007 10.1007/s10198-019-01119-1

[4] Celli BR, Decramer M, Wedzicha JA, Wilson KC, Agustí A, Criner GJ, et al. An Official American Thoracic Society/European Respiratory Society Statement: research questions in chronic obstructive pulmonary disease. Am J Respir Crit Care Med. 2015;191(7):e4–27. 25830527 10.1164/rccm.201501-0044ST

[5] Salvi SS, Barnes PJ. Chronic obstructive pulmonary disease in non-smokers. Lancet. 2009;374(9691):733–43. 19716966 10.1016/S0140-6736(09)61303-9

[6] Løkke A, Lange P, Scharling H, Fabricius P, Vestbo J. Developing COPD: a 25 year follow up study of the general population. Thorax. 2006;61(11):935–9. 17071833 10.1136/thx.2006.062802 PMC2121175

[7] Agustí A, Faner R. COPD beyond smoking: new paradigm, novel opportunities. Lancet Respir Med. 2018;6(5):324–6. 29496484 10.1016/S2213-2600(18)30060-2

[8] Cho MH, Hobbs BD, Silverman EK. Genetics of chronic obstructive pulmonary disease: understanding the pathobiology and heterogeneity of a complex disorder. Lancet Respir Med. 2022;10(5):485–96. 35427534 10.1016/S2213-2600(21)00510-5 PMC11197974

[9] Zhou JJ, Cho MH, Castaldi PJ, Hersh CP, Silverman EK, Laird NM. Heritability of chronic obstructive pulmonary disease and related phenotypes in smokers. Am J Respir Crit Care Med. 2013;188(8):941–7. 23972146 10.1164/rccm.201302-0263OC PMC3826281

[10] Soler Artigas M, Wain LV, Miller S, Kheirallah AK, Huffman JE, Ntalla I, et al. Sixteen new lung function signals identified through 1000 Genomes Project reference panel imputation. Nat Commun. 2015;6:8658. 26635082 10.1038/ncomms9658 PMC4686825

[11] Sakornsakolpat P, Prokopenko D, Lamontagne M, Reeve NF, Guyatt AL, Jackson VE, et al. Genetic landscape of chronic obstructive pulmonary disease identifies heterogeneous cell-type and phenotype associations. Nat Genet. 2019;51(3):494–505. 30804561 10.1038/s41588-018-0342-2 PMC6546635

[12] Shrine N, Izquierdo AG, Chen J, Packer R, Hall RJ, Guyatt AL, et al. Multi-ancestry genome-wide association analyses improve resolution of genes and pathways influencing lung function and chronic obstructive pulmonary disease risk. Nat Genet. 2023;55(3):410–22. 36914875 10.1038/s41588-023-01314-0 PMC10011137

[13] Foreman MG, Campos M, Celedón JC. Genes and chronic obstructive pulmonary disease. Med Clin North Am. 2012;96(4):699–711. 22793939 10.1016/j.mcna.2012.02.006 PMC3399759

[14] Shrine N, Guyatt AL, Erzurumluoglu AM, Jackson VE, Hobbs BD, Melbourne CA, et al. New genetic signals for lung function highlight pathways and chronic obstructive pulmonary disease associations across multiple ancestries. Nat Genet. 2019;51(3):481–93. 30804560 10.1038/s41588-018-0321-7 PMC6397078

[15] Billingsley H, Rodriguez-Miguelez P, Del Buono MG, Abbate A, Lavie CJ, Carbone S. Lifestyle interventions with a focus on nutritional strategies to increase cardiorespiratory fitness in chronic obstructive pulmonary disease, heart failure, obesity, sarcopenia, and frailty. Nutrients. 2019;11(12):2849. 31766324 10.3390/nu11122849 PMC6950118

[16] Fisher JE, Loft S, Ulrik CS, Raaschou-Nielsen O, Hertel O, Tjønneland A, et al. Physical activity, air pollution, and the risk of asthma and chronic obstructive pulmonary disease. Am J Respir Crit Care Med. 2016;194(7):855–65. 27653737 10.1164/rccm.201510-2036OC

[17] Nolasco T, Figueiredo R, Zanella P, Porcher F, Gass R, Hauck M, et al. Combined physical exercise in pulmonary rehabilitation does not alter endothelial function and vascular structure in chronic obstructive pulmonary disease: a randomized clinical trial. J Cardiopulm Rehabil Prev. 2025;45(3):215–23. 40167498 10.1097/HCR.0000000000000940

[18] Rasch-Halvorsen Ø, Hassel E, Langhammer A, Brumpton BM, Steinshamn S. The association between dynamic lung volume and peak oxygen uptake in a healthy general population: the HUNT study. BMC Pulm Med. 2019;19(1):2. 30612551 10.1186/s12890-018-0762-x PMC6322288

[19] Chen Z, Collings PJ, Wang M, Shi Q, Luo S, Au Yeung SL, et al. Physical fitness, biological aging, and healthy longevity. J Am Med Dir Assoc. 2025;26(10):105792. 40789340 10.1016/j.jamda.2025.105792

[20] Kunutsor SK, Jae SY, Mäkikallio TH, Laukkanen JA. Cardiorespiratory fitness does not offset the increased risk of chronic obstructive pulmonary disease attributed to smoking: a cohort study. Eur J Epidemiol. 2022;37(4):423–8. 35122562 10.1007/s10654-021-00835-4 PMC9187537

[21] Kunutsor SK, Laukkanen T, Laukkanen JA. Cardiorespiratory fitness is associated with reduced risk of respiratory diseases in middle-aged Caucasian men: a long-term prospective cohort study. Lung. 2017;195(5):607–11. 28698945 10.1007/s00408-017-0039-9

[22] Hansen GM, Marott JL, Holtermann A, Gyntelberg F, Lange P, Jensen MT. Midlife cardiorespiratory fitness and the long-term risk of chronic obstructive pulmonary disease. Thorax. 2019;74(9):843–8. 31209150 10.1136/thoraxjnl-2018-212821

[23] Kunutsor SK, Jae SY, Mäkikallio TH, Laukkanen JA. Cardiorespiratory fitness, inflammation, and risk of chronic obstructive pulmonary disease in middle-aged men: a cohort study. J Cardiopulm Rehabil Prev. 2022;42(5):347–51. 35121704 10.1097/HCR.0000000000000674

[24] Watts EL, Gonzales TI, Strain T, Saint-Maurice PF, Bishop DT, Chanock SJ, et al. Observational and genetic associations between cardiorespiratory fitness and cancer: a UK Biobank and international consortia study. Br J Cancer. 2024;130(1):114–24. 38057395 10.1038/s41416-023-02489-3 PMC10781786

[25] Cai L, Gonzales T, Wheeler E, Kerrison ND, Day FR, Langenberg C, et al. Causal associations between cardiorespiratory fitness and type 2 diabetes. Nat Commun. 2023;14(1):3904. 37400433 10.1038/s41467-023-38234-w PMC10318084

[26] Bycroft C, Freeman C, Petkova D, Band G, Elliott LT, Sharp K, et al. The UK Biobank resource with deep phenotyping and genomic data. Nature. 2018;562(7726):203–9. 30305743 10.1038/s41586-018-0579-z PMC6786975

[27] UK Biobank Group. UKB data showcase homepage. [Available from: https://biobank.ndph.ox.ac.uk/showcase/].

[28] Gonzales TI, Westgate K, Strain T, Hollidge S, Jeon J, Christensen DL, et al. Cardiorespiratory fitness assessment using risk-stratified exercise testing and dose-response relationships with disease outcomes. Sci Rep. 2021;11(1):15315. 34321526 10.1038/s41598-021-94768-3 PMC8319417

[29] Kokkinos P, Myers J, Franklin B, Narayan P, Lavie CJ, Faselis C. Cardiorespiratory fitness and health outcomes: a call to standardize fitness categories. Mayo Clin Proc. 2018;93(3):333–6. 29174511 10.1016/j.mayocp.2017.10.011

[30] Dudbridge F. Power and predictive accuracy of polygenic risk scores. PLoS Genet. 2013;9(3):e1003348. 23555274 10.1371/journal.pgen.1003348 PMC3605113

[31] Chang CC, Chow CC, Tellier LC, Vattikuti S, Purcell SM, Lee JJ. Second-generation PLINK: rising to the challenge of larger and richer datasets. Gigascience. 2015;4:7. 25722852 10.1186/s13742-015-0047-8 PMC4342193

[32] Zhou W, Kanai M, Wu KH, Rasheed H, Tsuo K, Hirbo JB, et al. Global biobank meta-analysis initiative: powering genetic discovery across human disease. Cell Genom. 2022;2(10):100192. 36777996 10.1016/j.xgen.2022.100192 PMC9903716

[33] Hanscombe KB, Persyn E, Traylor M, Glanville KP, Hamer M, Coleman JRI, et al. The genetic case for cardiorespiratory fitness as a clinical vital sign and the routine prescription of physical activity in healthcare. Genome Med. 2021;13(1):180. 34753499 10.1186/s13073-021-00994-9 PMC8579601

[34] Davies NM, Holmes MV, Davey Smith G. Reading Mendelian randomisation studies: a guide, glossary, and checklist for clinicians. BMJ. 2018;362:k601. 30002074 10.1136/bmj.k601 PMC6041728

[35] Burgess S, Thompson SG. Avoiding bias from weak instruments in Mendelian randomization studies. Int J Epidemiol. 2011;40(3):755–64. 21414999 10.1093/ije/dyr036

[36] Bowden J, Del Greco MF, Minelli C, Davey Smith G, Sheehan NA, Thompson JR. Assessing the suitability of summary data for two-sample Mendelian randomization analyses using MR-Egger regression: the role of the I2 statistic. Int J Epidemiol. 2016;45(6):1961–74. 27616674 10.1093/ije/dyw220 PMC5446088

[37] Bowden J, Davey Smith G, Burgess S. Mendelian randomization with invalid instruments: effect estimation and bias detection through Egger regression. Int J Epidemiol. 2015;44(2):512–25. 26050253 10.1093/ije/dyv080 PMC4469799

[38] Bowden J, Davey Smith G, Haycock PC, Burgess S. Consistent estimation in mendelian randomization with some invalid instruments using a weighted median estimator. Genet Epidemiol. 2016;40(4):304–14. 27061298 10.1002/gepi.21965 PMC4849733

[39] Karageorgiou V, Tyrrell J, McKinley TJ, Bowden J. Weak and pleiotropy robust sex-stratified Mendelian randomization in the one sample and two sample settings. Genet Epidemiol. 2023;47(2):135–51. 36682072 10.1002/gepi.22512

[40] Slob EAW, Burgess S. A comparison of robust Mendelian randomization methods using summary data. Genet Epidemiol. 2020;44(4):313–29. 32249995 10.1002/gepi.22295 PMC7317850

[41] Verbanck M, Chen CY, Neale B, Do R. Detection of widespread horizontal pleiotropy in causal relationships inferred from Mendelian randomization between complex traits and diseases. Nat Genet. 2018;50(5):693–8. 29686387 10.1038/s41588-018-0099-7 PMC6083837

[42] Hemani G, Zheng J, Elsworth B, Wade KH, Haberland V, Baird D, et al. The MR-base platform supports systematic causal inference across the human phenome. Elife. 2018. 10.7554/eLife.34408. 29846171 10.7554/eLife.34408 PMC5976434

[43] Vainshelboim B, Lima RM, Kokkinos P, Myers J. Cardiorespiratory fitness, lung cancer incidence, and cancer mortality in male smokers. Am J Prev Med. 2019;57(5):659–66. 31564605 10.1016/j.amepre.2019.04.020

[44] Kokkinos P, Faselis C, Franklin B, Lavie CJ, Sidossis L, Moore H, et al. Cardiorespiratory fitness, body mass index and heart failure incidence. Eur J Heart Fail. 2019;21(4):436–44. 30779281 10.1002/ejhf.1433

[45] Lin X, Zhang X, Guo J, Roberts CK, McKenzie S, Wu WC, et al. Effects of exercise training on cardiorespiratory fitness and biomarkers of cardiometabolic health: a systematic review and meta-analysis of randomized controlled trials. J Am Heart Assoc. 2015. 10.1161/JAHA.115.002014. 26116691 10.1161/JAHA.115.002014 PMC4608087

[46] Swift DL, Lavie CJ, Johannsen NM, Arena R, Earnest CP, O’Keefe JH, et al. Physical activity, cardiorespiratory fitness, and exercise training in primary and secondary coronary prevention. Circ J. 2013;77(2):281–92. 23328449 10.1253/circj.cj-13-0007 PMC7027930

[47] Chen N, Si X, Wang J, Chen W. Association of physical activity with asthma and chronic obstructive pulmonary disease and mediation of frailty: Mendelian randomization analyses. Int J Chron Obstruct Pulmon Dis. 2024;19:2309–20. 39429808 10.2147/COPD.S475714 PMC11491099

[48] Xiao L, Li W, Li F, Chen X, Xu Y, Hu Y, et al. Assessing the causal role of physical activity and leisure sedentary behaviours with chronic obstructive pulmonary disease: a Mendelian randomisation study. BMJ Open Respir Res. 2024. 10.1136/bmjresp-2023-001879. 38688688 10.1136/bmjresp-2023-001879 PMC11086375

[49] Brightling C, Greening N. Airway inflammation in COPD: progress to precision medicine. Eur Respir J. 2019. 10.1183/13993003.00651-2019. 31073084 10.1183/13993003.00651-2019

[50] Maestrelli P, Saetta M, Mapp CE, Fabbri LM. Remodeling in response to infection and injury. Airway inflammation and hypersecretion of mucus in smoking subjects with chronic obstructive pulmonary disease. Am J Respir Crit Care Med. 2001;164(10 Pt 2):S76-80. 11734472 10.1164/ajrccm.164.supplement_2.2106067

[51] Pauwels RA, Buist AS, Calverley PM, Jenkins CR, Hurd SS. Global strategy for the diagnosis, management, and prevention of chronic obstructive pulmonary disease. NHLBI/WHO global initiative for chronic obstructive lung disease (GOLD) workshop summary. Am J Respir Crit Care Med. 2001;163(5):1256–76. 11316667 10.1164/ajrccm.163.5.2101039

[52] Ertek S, Cicero A. Impact of physical activity on inflammation: effects on cardiovascular disease risk and other inflammatory conditions. Arch Med Sci. 2012;8(5):794–804. 23185187 10.5114/aoms.2012.31614 PMC3506236

[53] Hopkinson NS, Polkey MI. Does physical inactivity cause chronic obstructive pulmonary disease? Clin Sci Lond. 2010;118(9):565–72. 20132099 10.1042/CS20090458

