# Supplementary material for: Cardiorespiratory fitness, genetic susceptibility, and the risk of chronic obstructive pulmonary disease: findings from observational and two-sample bidirectional Mendelian randomisation analyses
Source: BMC Med. 2026 May 21;24:446. doi: 10.1186/s12916-026-04854-4 (PMC13480046; doi:10.1186/s12916-026-04854-4)
Supplement: Supplementary file 1 [file 12916_2026_4854_MOESM1_ESM.docx]

**Supplemental Material**

**Supplemental Table S1.** List of Single-Nucleotide Polymorphisms (SNPs) related to chronic obstructive pulmonary disease in White British ancestry.

**Supplemental Table S2.** Associations of genetic risk and sex- and age-standardised cardiorespiratory fitness with incident chronic obstructive pulmonary disease after excluding chronic obstructive pulmonary disease cases diagnosed within the first four years of follow-up.

**Supplemental Table S**3. Associations of sex- and age-standardised cardiorespiratory fitness with incident chronic obstructive pulmonary disease with further adjusting for behaviour factors (moderate and vigorous physical activity, TV viewing time and sleep duration).

**Supplemental Table S4**. Associations of sex- and age-standardised cardiorespiratory fitness with incident chronic obstructive pulmonary disease in genetically defined White British ancestry sample.

**Supplemental Table S5**. Associations of genetic risk and sex- and age-standardised cardiorespiratory fitness with incident chronic obstructive pulmonary disease after further excluding individuals with airflow obstruction (defined as a FEV1/FVC ratio <0.70) at baseline.

**Supplemental Table S6**. Associations of genetic risk and sex- and age-standardised cardiorespiratory fitness with algorithmically defined COPD.

**Supplemental Table S**7. Results of Mendelian randomisation between genetically predicted cardiorespiratory fitness and chronic obstructive pulmonary disease.

**Supplemental Figure S1.** Participants’ flow chart.

Table S1. A list of 71 Single-Nucleotide Polymorphism (SNPs) known to be associated with either chronic obstructive pulmonary disease or lung function for individuals of White British ancestry with genome-wide significance at a p-value of 5×10^-8^ and in low linkage disequilibrium defined according to r^2^<0.001.

| SNP | Gene / nearest gene | Effect allele | Other allele | Effect allele frequency | P-value | *Beta* |
| --- | --- | --- | --- | --- | --- | --- |
| rs9435731 | MFAP2 | A | C | 0.51 | 6.60E-10 | 0.058269 |
| rs76841360 | HEYL/PABPC4 | A | G | 0.23 | 5.00E-10 | 0.076961 |
| rs11118406 | SLC30A10 | T | A | 0.28 | 4.10E-11 | 0.076961 |
| rs11579382 | CHRM3 | C | G | 0.42 | 6.50E-09 | 0.058269 |
| rs72902175 | LINC01876 | T | C | 0.13 | 1.60E-08 | 0.086178 |
| rs2571445 | TNS1 | A | G | 0.39 | 3.40E-12 | 0.067659 |
| rs16825267 | LINC01807 | C | G | 0.92 | 1.80E-20 | 0.173953 |
| rs62191105 | LINC01940 | C | T | 0.8 | 2.60E-12 | 0.086178 |
| rs1529672 | RARB | C | A | 0.83 | 2.50E-11 | 0.086178 |
| rs13073544 | RBMS3 | C | G | 0.28 | 2.00E-08 | 0.058269 |
| rs17759204 | LINC02017 | G | A | 0.27 | 8.80E-11 | 0.067659 |
| rs2955083 | EEFSEC | A | T | 0.88 | 3.50E-15 | 0.122218 |
| rs7642001 | LINC01997 | A | G | 0.37 | 1.10E-14 | 0.076961 |
| rs7671261 | FAM13A | A | G | 0.55 | 1.40E-18 | 0.086178 |
| rs34712979 | NPNT | A | G | 0.25 | 3.00E-46 | 0.165514 |
| rs13140176 | KRT18P51 | A | G | 0.61 | 4.10E-59 | 0.165514 |
| rs1551943 | ITGA1 | A | G | 0.23 | 5.80E-10 | 0.076961 |
| rs153916 | SPATA9 | T | C | 0.55 | 6.30E-10 | 0.058269 |
| rs10037493 | HTR4 | C | T | 0.55 | 2.60E-33 | 0.122218 |
| rs10866659 | ADAM19 | G | A | 0.35 | 1.20E-16 | 0.086178 |
| rs2806356 | ARMC2 | C | T | 0.18 | 2.90E-15 | 0.09531 |
| rs9399401 | ADGRG6 | T | C | 0.72 | 1.60E-40 | 0.14842 |
| rs2897075 | ZKSCAN1 | C | T | 0.63 | 7.30E-10 | 0.067659 |
| rs10114763 | GLIS3 | T | A | 0.42 | 8.70E-13 | 0.067659 |
| rs803923 | ASTN2 | A | G | 0.53 | 2.70E-08 | 0.058269 |
| rs7068966 | CDC123 | C | T | 0.49 | 6.20E-23 | 0.09531 |
| rs2579762 | LRMDA | C | A | 0.47 | 2.60E-10 | 0.058269 |
| rs117261012 | PRSS23 | G | A | 0.16 | 6.90E-10 | 0.086178 |
| rs11049386 | CCDC91 | T | A | 0.71 | 2.70E-08 | 0.058269 |
| rs7307510 | SNRPF-DT | C | T | 0.81 | 2.60E-09 | 0.076961 |
| rs72699855 | RIN3 | G | C | 0.81 | 4.80E-09 | 0.076961 |
| rs1441358 | THSD4 | G | T | 0.34 | 7.40E-33 | 0.122218 |
| rs55676755 | CHRNA3 | G | C | 0.34 | 2.70E-26 | 0.10436 |
| rs56134392 | EMP2 | C | T | 0.35 | 4.50E-08 | 0.058269 |
| rs4888379 | CFDP1 | T | A | 0.58 | 5.90E-21 | 0.09531 |
| rs34727469 | EPOP | T | C | 0.14 | 1.10E-08 | 0.086178 |
| rs12373142 | SPPL2C | G | C | 0.22 | 9.90E-10 | 0.076961 |
| rs11655567 | RNU7-155P | C | T | 0.49 | 1.90E-09 | 0.058269 |
| rs647097 | MTCL1 | C | T | 0.27 | 1.00E-11 | 0.076961 |
| rs2096468 | MRPS6 | A | C | 0.45 | 4.00E-08 | 0.058269 |
| rs9617650 | MICAL3 | G | C | 0.79 | 4.40E-10 | 0.076961 |
| rs72673419 | LINC02778 | T | C | 0.05 | 4.00E-09 | 0.131028 |
| rs629619 | DENND2D | T | C | 0.2 | 2.90E-10 | 0.076961 |
| rs4585380 | BTC | G | A | 0.74 | 3.40E-09 | 0.067659 |
| rs12519165 | SMIM23 | A | T | 0.38 | 1.10E-09 | 0.067659 |
| rs646695 | LINC02941 | C | T | 0.24 | 4.60E-11 | 0.076961 |
| rs2040732 | ITGB8 | C | T | 0.58 | 6.90E-09 | 0.058269 |
| rs1570221 | STN1 | A | G | 0.35 | 2.20E-08 | 0.058269 |
| rs4757118 | RASSF10 | T | C | 0.54 | 3.80E-09 | 0.058269 |
| rs9525927 | LINC02938 | G | A | 0.19 | 2.80E-09 | 0.076961 |
| rs72731149 | ATP8B4 | G | C | 0.91 | 8.30E-09 | 0.113329 |
| rs955277 | ASAP2 | T | C | 0.61 | 1.90E-10 | 0.067659 |
| rs12466981 | EML4 | C | T | 0.73 | 4.90E-08 | 0.058269 |
| rs2442776 | VGLL4 | G | A | 0.15 | 2.00E-10 | 0.086178 |
| rs4093840 | ADCY5 | A | T | 0.47 | 3.90E-10 | 0.058269 |
| rs979453 | CCDC69 | G | A | 0.34 | 1.40E-08 | 0.058269 |
| rs1334576 | RREB1 | A | G | 0.42 | 1.20E-08 | 0.058269 |
| rs9350191 | ID4 | T | C | 0.85 | 5.10E-14 | 0.113329 |
| rs2284174 | IER3 | C | T | 0.22 | 2.10E-21 | 0.113329 |
| rs674621 | RFX6 | C | T | 0.32 | 7.60E-09 | 0.058269 |
| rs9329170 | MFHAS1 | C | G | 0.86 | 3.60E-10 | 0.09531 |
| rs10760580 | ADIPINT | G | A | 0.71 | 1.20E-10 | 0.067659 |
| rs8044657 | SPMIP8 | G | A | 0.9 | 1.10E-08 | 0.10436 |
| rs4660861 | TESK2 | G | T | 0.57 | 4.40E-08 | 0.058269 |
| rs7650602 | ZBTB38 | C | T | 0.45 | 4.90E-08 | 0.058269 |
| rs34651 | TNPO1 | C | T | 0.08 | 3.00E-08 | 0.10436 |
| rs798565 | AMZ1 | G | A | 0.71 | 3.90E-09 | 0.067659 |
| rs7866939 | RPS6P12 | C | T | 0.33 | 1.70E-08 | 0.058269 |
| rs7958945 | UBA52P7 | G | A | 0.36 | 1.00E-09 | 0.058269 |
| rs72626215 | DMWD | G | A | 0.73 | 1.70E-09 | 0.067659 |
| rs73158393 | SYN3 | C | G | 0.74 | 7.70E-09 | 0.067659 |

Table S2. Associations of genetic risk and sex- and age-standardised cardiorespiratory fitness with incident chronic obstructive pulmonary disease after excluding chronic obstructive pulmonary disease cases diagnosed within the first four years of follow-up.

|  |  |  | Hazard ratio of COPD | |
| --- | --- | --- | --- | --- |
|  |  |  | (95% confidence intervals) | |
| Comparisons | No. of Participants | No. of events | Model 1 | Model 2 |
| Tertiles of polygenic risk |  |  |  |  |
| score for COPD |  |  |  |  |
| Low (Reference) | 22,541 | 478 | 1.00 (ref) |  |
| Medium | 22,538 | 568 | 1.18 (1.04-1.33) |  |
| High | 22,521 | 648 | 1.34 (1.19-1.33) |  |
| PRS-COPD (continuous) | 67,600 | 1,694 | 1.12 (1.07-1.18) |  |
| Tertiles of CRF |  |  |  |  |
| Low (Reference) | 22,544 | 710 | 1.00 (ref) | 1.00 (ref) |
| Medium | 22,548 | 532 | 0.79 (0.70-0.90) | 0.81 (0.72-0.92) |
| High | 22,508 | 452 | 0.72 (0.64-0.84) | 0.75 (0.65-0.86) |
| CRF (per 1-SD difference) | 67,600 | 1,694 | 0.88 (0.83-0.94) | 0.90 (0.85-0.95) |

Note:

The analyses were performed based on multivariable Cox proportional hazards models.

Model 1: Model for PRS-COPD adjusted for age, sex, genotyping array and the first twenty principal components of genetic ancestry. Model for CRF adjusted for age, sex, muscle strength (assess via grip strength for both hands), smoking status (never, previous, current), employment (unemployed, employed), Townsend Deprivation Index, alcohol consumption (never, previous, currently <3 times/week, currently >=3 times/week), body mass index, history of diabetes, history of hypertension, history of asthma, and air-pollution exposure (assessed based on residential levels of PM₂.₅, PM₁₀, and nitrogen dioxide)

Model 2: adjusted for all confounders in model 2 with an additional adjustment for PRS-COPD, genotyping array and first twenty principal components of ancestry.

Abbreviations: COPD, chronic obstructive pulmonary disease. CRF, cardiorespiratory fitness. PRS-COPD, polygenic risk score for chronic obstructive pulmonary disease

Table S3. Associations of sex- and age-standardised cardiorespiratory fitness with incident chronic obstructive pulmonary disease with further adjusting for behaviour factors (moderate and vigorous physical activity, TV viewing time and sleep duration).

|  |  |  | Hazard ratio of COPD | |
| --- | --- | --- | --- | --- |
|  |  |  | (95% confidence intervals) | |
| Comparisons | No. of Participants | No. of events | Model 1 | Model 2 |
| Tertiles of CRF |  |  |  |  |
| Low (Reference) | 20,229 | 675 | 1.00 (ref) | 1.00 (ref) |
| Medium | 20,184 | 492 | 0.75 (0.67-0.85) | 0.77 (0.68-0.87) |
| High | 20,186 | 424 | 0.68 (0.59-0.79) | 0.70 (0.61-0.81) |
| CRF (per 1-SD difference) | 60,599 | 1,591 | 0.86 (0.81-0.92) | 0.88 (0.82-0.93) |

Note:

The analyses were performed based on multivariable Cox proportional hazards models.

Model 1: adjusting for age, sex, muscle strength (assess via grip strength for both hands), smoking status (never, previous, current), employment (unemployed, employed), Townsend Deprivation Index, alcohol consumption (never, previous, currently <3 times/week, currently >=3 times/week) , air-pollution exposure (assessed based on residential levels of PM₂.₅, PM₁₀, and nitrogen dioxide), body mass index, history of diabetes, history of hypertension, history of asthma, air-pollution exposure (assessed based on residential levels of PM₂.₅, PM₁₀, and nitrogen dioxide), moderate and vigorous physical activity (minute/day), TV viewing time (hours/day), and sleep duration (<7 hours/day, 7-9 h/ hours/day, >9 h hours /day)

Model 2: adjusting for all confounders in model 2 with an additional adjustment for PRS-COPD, genotyping array and first twenty principal components of ancestry.

Abbreviations: COPD, chronic obstructive pulmonary disease. CRF, cardiorespiratory fitness. PRS-COPD, polygenic risk score for chronic obstructive pulmonary disease.

Table S4. Associations of genetic risk and sex- and age-standardised cardiorespiratory fitness with incident chronic obstructive pulmonary disease in genetically defined White British ancestry sample.

|  |  |  | Hazard ratio of COPD | |
| --- | --- | --- | --- | --- |
|  |  |  | (95% confidence intervals) | |
| Comparisons | No. of Participants | No. of events | Model 1 | Model 2 |
| Tertiles of polygenic risk |  |  |  |  |
| score for COPD |  |  |  |  |
| Low (Reference) | 19,929 | 489 | 1.00 (ref) |  |
| Medium | 19,831 | 583 | 1.19 (1.06-1.35) |  |
| High | 19,865 | 648 | 1.31 (1.17-1.48) |  |
| PRS-COPD (continuous) | 59,625 | 1,720 | 1.12 (1.07-1.17) |  |
| Tertiles of CRF |  |  |  |  |
| Low (Reference) | 19,902 | 722 | 1.00 (ref) | 1.00 (ref) |
| Medium | 19,867 | 550 | 0.79 (0.71-0.89) | 0.82 (0.73-0.92) |
| High | 19,856 | 448 | 0.68 (0.60-0.77) | 0.70 (0.62-0.81) |
| CRF (per 1-SD difference) | 59,625 | 1,720 | 0.86 (0.82-0.90) | 0.88 (0.83-0.93) |

Note:

The analyses were performed based on multivariable Cox proportional hazards models.

Model 1: Model for PRS-COPD adjusted for age, sex, genotyping array and the first twenty principal components of genetic ancestry. Model for CRF adjusted for age, sex, muscle strength (assess via grip strength for both hands), smoking status (never, previous, current), employment (unemployed, employed), Townsend Deprivation Index, alcohol consumption (never, previous, currently <3 times/week, currently >=3 times/week), body mass index, history of diabetes, history of hypertension, history of asthma, and air-pollution exposure (assessed based on residential levels of PM₂.₅, PM₁₀, and nitrogen dioxide)

Model 2: adjusted for all confounders in model 2 with an additional adjustment for PRS-COPD, genotyping array and first twenty principal components of ancestry.

Abbreviations: COPD, chronic obstructive pulmonary disease. CRF, cardiorespiratory fitness. PRS-COPD, polygenic risk score for chronic obstructive pulmonary disease

Table S5. Associations of genetic risk and sex- and age-standardised cardiorespiratory fitness with incident chronic obstructive pulmonary disease after further excluding individuals with airflow obstruction (defined as a FEV1/FVC ratio <0.70) at baseline.

|  |  |  | Hazard ratio of COPD | |
| --- | --- | --- | --- | --- |
|  |  |  | (95% confidence intervals) | |
| Comparisons | No. of Participants | No. of events | Model 1 | Model 2 |
| Tertiles of polygenic risk |  |  |  |  |
| score for COPD |  |  |  |  |
| Low (Reference) | 19,416 | 329 | 1.00 (ref) |  |
| Medium | 19,417 | 372 | 1.28 (1.11-1.50) |  |
| High | 19,397 | 380 | 1.30 (1.15-1.53) |  |
| PRS-COPD (continuous) | 58,230 | 1,053 | 1.09 (1.03-1.16) |  |
| Tertiles of CRF |  |  |  |  |
| Low (Reference) | 19,432 | 477 | 1.00 (ref) | 1.00 (ref) |
| Medium | 19,390 | 337 | 0.76 (0.66-0.87) | 0.77 (0.66-0.90) |
| High | 19,408 | 239 | 0.60 (0.50-0.72) | 0.61 (0.51-0.73) |
| CRF (per 1-SD difference) | 58,230 | 1,053 | 0.80 (0.74-0.86) | 0.81 (0.75-0.88) |

Note:

The analyses were performed based on multivariable Cox proportional hazards models.

Model 1: Model for PRS-COPD adjusted for age, sex, genotyping array and the first twenty principal components of genetic ancestry. Model for CRF adjusted for age, sex, muscle strength (assess via grip strength for both hands), smoking status (never, previous, current), employment (unemployed, employed), Townsend Deprivation Index, alcohol consumption (never, previous, currently <3 times/week, currently >=3 times/week), body mass index, history of diabetes, history of hypertension, history of asthma, and air-pollution exposure (assessed based on residential levels of PM₂.₅, PM₁₀, and nitrogen dioxide)

Model 2: adjusted for all confounders in model 2 with an additional adjustment for PRS-COPD, genotyping array and first twenty principal components of ancestry.

Abbreviations: COPD, chronic obstructive pulmonary disease. CRF, cardiorespiratory fitness. PRS-COPD, polygenic risk score for chronic obstructive pulmonary disease

Table S6. Associations of genetic risk and sex- and age-standardised cardiorespiratory fitness with algorithmically defined COPD.

|  |  |  | Hazard ratio of COPD | |
| --- | --- | --- | --- | --- |
|  |  |  | (95% confidence intervals) | |
| Comparisons | No. of Participants | No. of events | Model 1 | Model 2 |
| Tertiles of polygenic risk |  |  |  |  |
| score for COPD |  |  |  |  |
| Low (Reference) | 22,129 | 542 | 1.00 (ref) |  |
| Medium | 21,991 | 629 | 1.14 (1.02-1.29) |  |
| High | 21,918 | 719 | 1.32 (1.18-1.47) |  |
| PRS-COPD (continuous) | 67,928 | 1,890 | 1.12 (1.06-1.17) |  |
| Tertiles of CRF |  |  |  |  |
| Low (Reference) | 21,882 | 790 | 1.00 (ref) | 1.00 (ref) |
| Medium | 22,024 | 594 | 0.82 (0.73-0.92) | 0.84 (0.74-0.94) |
| High | 22,132 | 506 | 0.74 (0.65-0.85) | 0.76 (0.67-0.87) |
| CRF (per 1-SD difference) | 67,928 | 1,890 | 0.88 (0.84-0.94) | 0.90 (0.85-0.95) |

Note:

The analyses were performed based on multivariable Cox proportional hazards models. COPD incident cases were defined using algorithmically-defined outcomes (Data-fields 42016)。

Model 1: Model for PRS-COPD adjusted for age, sex, genotyping array and the first twenty principal components of genetic ancestry. Model for CRF adjusted for age, sex, muscle strength (assess via grip strength for both hands), smoking status (never, previous, current), employment (unemployed, employed), Townsend Deprivation Index, alcohol consumption (never, previous, currently <3 times/week, currently >=3 times/week), body mass index, history of diabetes, history of hypertension, history of asthma, and air-pollution exposure (assessed based on residential levels of PM₂.₅, PM₁₀, and nitrogen dioxide)

Model 2: adjusted for all confounders in model 2 with an additional adjustment for PRS-COPD, genotyping array and first twenty principal components of ancestry.

Abbreviations: COPD, chronic obstructive pulmonary disease. CRF, cardiorespiratory fitness. PRS-COPD, polygenic risk score for chronic obstructive pulmonary disease

Table S7. Results of Mendelian randomisation using 160 combined SNPs for fitness (cardiorespiratory fitness and resting heart rate) on chronic obstructive pulmonary disease.

| Method | Number of SNPs | Odds ratio  (95% CI) | p-value | Cochran’s Q *p*-value | Egger Intercept p-value |
| --- | --- | --- | --- | --- | --- |
| Inverse-variance weighted | 94 | 0.98 (0.97-0.99) | **0.001** | 0.78 |  |
| Weighted median | 94 | 0.99 (0.96-1.005) | 0.161 |  |  |
| MR Egger | 94 | 0.98 (0.96-1.002) | 0.083 |  | 0.82 |
| Robust adjusted profile score | 94 | 0.98 (0.97-0.99) | **0.002** |  |  |
| MR-PRESSO | 94 | 0.98 (0.97-0.99) | **0.010** |  |  |

Note: Outlier SNPs were excluded using MR-PRESSO method before MR analyses.^40^ After excluding the outlier, 94 SNPs were included in both CRF and COPD GWAS results. The genetic instruments on chronic obstructive pulmonary disease were extracted from the meta-analysis of GWAS summary statistics from a Global Biobank study in European ancestry (58,559 cases and 937,358 controls) [31]. The effect estimates on cardiorespiratory fitness were extracted from meta-analysis summary statistics among European [25].

Figure S1. Participants’ flow chart.
